# Education, economic autonomy and digitalization as factors associated with married women’s ability to make sexual and reproductive health decisions in sub-Saharan Africa: a multi-level analysis of 16 countries

**DOI:** 10.1186/s12905-025-03944-4

**Published:** 2025-08-26

**Authors:** Turnwait Otu Michael, Kammila Naidoo

**Affiliations:** Department of Sociology, University of Johannesburg, Johannesburg, South Africa

**Keywords:** Educational access, Economic autonomy, Digitalization, Decision-making ability, Sexual and reproductive health

## Abstract

**Background:**

Despite progress in various aspects of women’s rights and health, a significant gap persists in the area of reproductive health decisions among women in Sub-Saharan Africa. This study seeks to address this gap by investigating the interplay between educational access, economic autonomy, and digital systems on the sexual and reproductive health (SRH) outcomes of married women in the Sub-Saharan African context.

**Methods:**

Leveraging cross-sectional data obtained from the latest demographic and health surveys conducted across 16 Sub-Saharan African nations between January 2015 and December 2021, the study’s focus centred on 67,437 married women. Through the utilization of binary logistic regression, model-adjusted odds ratios accompanied by a 95% confidence interval were calculated.

**Results:**

Compared to their counterparts in Benin, married women in Madagascar (aOR = 4.95, 95% CI = 4.29–5.71) and Uganda (aOR = 3.42, 95% CI = 2.99–3.90) exhibited the highest levels of agency in making SRH decisions among the 16 countries studied. Factors like higher education (aOR = 2.37, 95% CI = 2.13–2.64), familiarity with modern contraception (aOR = 3.19, 95% CI = 1.99–5.13), utilization of mobile phones for financial transactions (aOR = 1.21, 95% CI = 1.14–1.28), possession of a bank account (aOR = 1.17, 95% CI = 1.10–1.25), current employment (aOR = 1.19, 95% CI = 1.08–1.32), and engagement with the internet (aOR = 1.16, 95% CI = 1.08–1.24) were found to significantly influence SRH decisions-making across all countries.

**Conclusions:**

Enhancing the sexual and reproductive health outcomes of married women in Sub-Saharan Africa necessitates targeted efforts, particularly among those without higher education, limited contraceptive knowledge, reliance on mobile phones for financial transactions, without bank accounts, lacking internet access, and currently unemployed. By addressing these specific areas, interventions can be more effective in bridging the existing gaps in SRH decision-making among married women in this region.

## Background

The enhancement of women’s decision-making prowess, financial autonomy, and open discourse on sexual and reproductive health (SRH) hinges on pivotal factors like education, economic self-sufficiency, and digitalization [1–4]. To eliminate social and cultural barriers that impede women’s SRH progress, empowering measures such as education, economic advancement, and digital integration take centre stage [5, 6]. Recent global attention has been galvanized around women’s education and financial self-reliance [7]. Importantly, the attainment of UN Sustainable Development Goals (SDGs) requires the concerted prioritization of married women’s education and economic empowerment —financial independence and decision-making ability— particularly in the current phase of the Fourth Industrial Revolution (4IR) and digital revolution catalysed by information and communication technologies (ICTs) [8, 9].

Consequently, international and regional entities, alongside networks, have rallied for digital advocacy to embrace women’s inclusion. For instance, the Mexico City Consensus of 2004 passionately advocated for “enabling all women’s access to information and communication technologies” [10]. Equally noteworthy, the twelfth Regional Conference on Women in Latin America and the Caribbean in 2013 underscored the pivotal role of nurturing women’s education within the digital economy to quell gender disparities and pave the way for sustainable development [10]. Echoing this gush, UN Women emphatically declared in 2023 that “digital rights are women’s rights” [7], and in 2025 called “for all women and girls: rights, equality, and empowerment” as catalysts for lasting change [11]. It is indisputable that harnessing digitization’s potential, coupled with evidence-based data transformation, is a fundamental imperative to uplift married women’s education, financial status, and reproductive health in our contemporary society [12–14].

Despite its potential, digitalization—a transformative journey leveraging digital technology for enhancing business transactions and decisions—does carry inherent limitations such as unequal access to digital resources, privacy concerns, and the potential for misinformation, which can exacerbate existing inequalities in SRH decision-making [1, 15, 16]. Yet, researchers retain a buoyant outlook, positing that adept regulation and balanced implementation can mitigate digitalization’s adverse effects and usher in a promising era of swift socio-economic advancement [13, 17]. This emphasizes the interplay between digitalization, married women’s education, economic autonomy, and SRH, influencing a plethora of sectors [18, 19]. Consequently, engineering innovative solutions and developments to uplift married women’s SRH necessitates dismantling gender inequality, offering equitable opportunities for all [20, 21]. In this mission, the glaring gender divide requires focused attention, particularly as women comprise nearly half of the global population—49.58% [22, 23]—yet encounter restricted access to ICTs [7, 24].

Progress has indeed been registered in addressing gender disparities within developed nations, albeit with varying degrees [25, 26]. However, in developing countries, the gender imbalance remains entrenched across all domains [2, 27]. The sub-Saharan African (SSA) landscape, in particular, witnesses married women lagging behind in earnings, asset ownership, bank account utilization, and control over their income. In this region, numerous married women lack essential assets like land and buildings [28, 29], often navigating their business transactions through their husbands’ accounts due to prevailing patriarchal norms [28, 30]. As a result, this structural constraint hampers women’s trading and investment pursuits [31, 32].

Although stakeholders at different levels (federal, state, and local governments) have undertaken policies and programs to underscore their commitment to women’s education, economic autonomy, and digital integration, a substantial gender divide persists across SSA [2, 33]. Hence, a compelling impetus exists for empirical research that scrutinizes the gender gap in the region, particularly when it comes to female-focused initiatives. To amplify advocacy efforts and guide well-informed policy decisions, the present study delves into the nexus between education, economic empowerment, digitalization, and married women’s SRH. This exploration draws from the wealth of data gleaned from demographic and health surveys conducted among a diverse group of women across 16 countries in SSA.

## Methods

### Data source

The data utilized in this study were drawn from the datasets of the Demographic and Health Surveys (DHS). The DHS is a comprehensive, nationally representative cross-sectional survey conducted in over 90 countries at five-year intervals. Notably, it encompasses various SSA countries and other developing nations. These surveys gathered data from women within the reproductive age bracket of 15 to 49. The current study exclusively utilized surveys carried out in SSA countries. The dataset was restricted to the most recent surveys conducted in 16 SSA nations spanning from January 2015 to December 2021. These 16 nations were deliberately selected due to the alignment of their DHS statistics with our specific variables of interest, which gauge women’s education, economic autonomy, digitalization, and SRH matters. All these countries employed the latest DHS Model Questionnaire - Phase 7 to collect data on digital banking ownership and usage, internet access, and mobile phone utilization for business transactions. The data were sourced from individual recode files specific to women.

The DHS employed a multi-stage sampling method for data collection across countries. This commenced with cluster sampling to select enumeration areas (EAs), followed by systematic sampling for households, and finally, simple random sampling to choose respondents for questionnaire administration. For this study, only married women were included in the databases. The sample encompassed 67,437 married women respondents who satisfied the inclusion criteria of being married and providing responses to all questions related to our focal variables. Responses of ‘don’t know’ or ‘not sure’ led to exclusion from our married women sample. The distribution of the sample across the chosen SSA is presented in Table 1. During the drafting of this manuscript, the authors adhered to the ‘Strengthening the Reporting of Observational Studies in Epidemiology’ (STROBE) statement guidelines [34].

**Table 1:** Sample size and year of survey

Country	Year	Weighted Sample	Weighted %
1. Benin	2017	2666	4.0
2. Burundi	2016-17	2063	3.1
3. Ethiopia	2016	1955	2.9
4. Gambia	2019-20	5498	8.2
5. Guinea	2018	4416	6.5
6. Liberia	2019-20	1641	2.4
7. Madagascar	2021	3441	5.1
8. Mali	2018	4642	6.9
9. Malawi	2018	5139	7.6
10. Nigeria	2018	14,309	21.2
11. Rwanda	2019-20	3312	4.9
12. Sierra Leone	2019	3677	5.5
13. Uganda	2016	4677	6.9
14. South Africa	2016	2587	3.8
15. Zambia	2018	3699	5.5
16. Zimbabwe	2015	3712	5.5
All Countries	-	67,437	100

Source: Demographic and Health Surveys

### Study variables

#### Outcome variable

The primary outcome variable in this study is married women’s ability to make decisions regarding their sexual and reproductive health (SRH). This was assessed through four specific questions:

1. “Can you refuse or say no to sex with your husband/partner if you do not want to have sex?”
2. “Can you request that your husband/partner uses a condom?”
3. “Who usually decides whether or not to use contraception?”
4. “Who usually decides on your health care?”

Responses to the first two questions were coded as “1 = yes” and “0 = no,” indicating whether the woman could refuse sexual activity or request condom use. Responses to the latter two questions on contraception and healthcare decision-making were categorized as follows: “1 = respondent alone,” “2 = husband/partner alone,” and “3 = joint decision (both decide together).” These responses were further recoded into a binary format where “1 = respondent involved in decision-making” and “0 = respondent not involved in decision-making.” A woman was considered to have SRH autonomy if she was either independently or jointly involved in these decisions. Conversely, she lacks reproductive autonomy when she is entirely excluded from the decision-making process [35]. The final outcome variable was derived as a dichotomous measure: “1 = married women’s ability to make SRH decisions” and “0 = married women’s inability to make SRH decisions.” The binary format required for the logistic regression analysis was derived from the outcome responses coded ‘1’ and ‘0’.

#### Independent variables

A total of 14 independent variables were included in this study, capturing various dimensions of education, economic autonomy, and digitalization. These variables and their categorizations are detailed in Table 2.

**Table 2:** Study variables and their categories

Variable	Category and Coding
Education Level	No education (0), Primary (1), Secondary (2), Higher (3)
Knowledge of Contraceptive Methods	Knows no method (0), Folkloric (1), Traditional (2), Modern (3)
Media Engagement	Radio listening: Not at all (0), < 1 week (1), 1 + week (2), Daily (3)
	TV watching: Not at all (0), < 1 week (1), 1 + week (2), Daily (3)
Digital Tool Utilization	Uses mobile phone for financial transactions: No (0), Yes (1)
	Has a bank/financial institution account: No (0), Yes (1)
	Internet usage: Never (0), Last 12 months (1), Before last 12 months (2), Used but timing unclear (3)
Economic Autonomy	Currently working: No (0), Yes (1)
	Decision-maker on respondent’s earnings: Self (1), Self & Partner (2), Self & Other (3), Partner alone (4), Someone else (5)
	Land ownership: No ownership (0), Sole ownership (1), Joint ownership (2), Both sole & joint (3)
Wealth Index	Poorest (1), Poorer (2), Middle (3), Richer (4), Richest (5)
Residency	Urban (1), Rural (2)
Age Groups	15–19 (1), 20–24 (2), 25–29 (3), 30–34 (4), 35–39 (5), 40–44 (6), 45–49 (7)
Country of Survey	Refer to Table 1 for details

In evaluating the wealth index, the DHS utilized a Principal Component Analysis (PCA) approach to continuously measure household assets, encompassing items like cars, bicycles, televisions, building materials, sanitation facilities, and water sources [36]. This continuous measure was then transformed into a wealth quintile, categorized into five groups. The selection of independent variables was guided by their availability within the DHS databases and informed by existing literature, underpinning their relevance for this study’s exploration.

### Statistical analyses

The study data underwent analysis across three tiers. Initially, a univariate analysis was executed, encompassing frequencies and percentages. This initial step delved into the study sample in terms of the survey year and country, respondent demographics, as well as contextual variables such as those probing education, women’s economic autonomy, and digitalization. To mitigate potential sampling errors, the sample was weighted using the DHS recommended syntax command for individual women’s recode file unit of analysis (v005) via SPSS statistics. Omissions or missing in terms of both user and system variables were removed to counteract confounding variable effects.

The second level encompassed bivariate analysis, involving cross-tabulation and the chi-square test. This step aimed to establish associations between the dependent variable and each independent variable, considering a *p*-value threshold of less than 0.05. Only variables demonstrating significance at the bivariate stage progressed to the subsequent multivariate level. These variables were subjected to a collinearity diagnostic assessment to ensure they met multicollinearity prerequisites and were devoid of collinearity errors. Results indicated the absence of collinearity among the explanatory variables, with variance inflation factor (VIF) values ranging between 1.02 and 1.40, substantiating their suitability for advanced analysis.

The third level entailed a comprehensive analysis of predictive impacts. Binary logistic regression was employed across five models to measure the independent variables’ predictive effects on married women’s ability to make SRH decisions in SSA. Model 1 examined country-level variations in women’s SRH decision-making capacity in isolation from other explanatory factors. Model II delved into the influence of education on SRH decision-making capacity, focusing solely on education level components. Model III explored the impact of digitalization variables on SRH decision-making ability in isolation. Model IV scrutinized the connection between women’s economic autonomy and SRH decision-making capacity, independent of other factors. The conclusive Model V encompassed the entire array of predictive variables, both individual and contextual. Adjusted odds ratios (aOR) with 95% confidence intervals were reported for each model. Reference categories were determined based on groups with the lowest likelihood of making SRH decisions within marriage, aligned with existing literature. SPSS Statistics v25 facilitated all data analysis procedures.

## Results

### Background characteristics and sexual and reproductive health decision

Table 3 presents the associations of various independent factors on the participation of married women in SRH decisions. Notably, higher education (53%) and familiarity with modern contraceptive methods (35%) were associated with greater involvement in SRH decisions among married women. Frequent radio listeners (48.6%) and avid television viewers (52.4%) exhibited heightened engagement in the SRH decision-making process. The utilization of mobile phones for financial transactions (47.7%), possession of a bank or financial institution account (48.8%), and recent internet usage within the past 12 months (40.8%) were linked to increased participation in SRH decisions. Furthermore, married women presently engaged in work (37.3%), those co-deciding expenditure with their husbands (52.3%), and those holding joint ownership of land (40.2%) demonstrated elevated involvement in SRH decisions. The wealthiest wealth index category (43.7%), urban residency (37.5%), and the age group 35–39 exhibited higher participation in SRH decision-making. All independent variables exhibited significant associations with married women’s participation in SRH decisions in SSA, as confirmed by chi-square analysis.

**Table 3:** Involvement of married women in sexual and reproductive health decisions at various levels of independent variables, weighted *N* = 67,437

Variable	Involved in Sexual and Reproductive Health Decisions				
	No		Yes		X^2^ (*p*-value)
	Frequency (*N* = 44,172)	%	Frequency (*N* = 23,265)	%	
Education					4924.3 (< 0.001)
No education	14,794	86.2	2368	13.8	
Primary	11,252	62.9	6638	37.1	
Secondary	14,850	58.4	10,569	41.6	
Higher	3277	47.0	3691	53.0	
Knowledge of contraceptive method					441.7 (< 0.001)
Knows no method	973	95.8	43	4.2	
Knows only folkloric method	34	94.4	2	5.6	
Knows only traditional method	49	84.5	9	15.5	
Knows modern method	43,115	65.0	23,211	35.0	
Frequency of listening to radio					614.7 (< 0.001)
Not at all	13,983	71.5	5571	28.5	
Less than once a week	10,894	66.5	5480	33.5	
At least once a week	18,813	61.5	11,759	38.5	
Almost every day	482	51.4	455	48.6	
Frequency of watching television					532.9 (< 0.001)
Not at all	19,641	69.0	8822	31.0	
Less than once a week	8092	66.8	4016	33.2	
At least once a week	15,631	62.1	9538	37.9	
Almost every day	808	47.6	889	52.4	
Use mobile phone for financial transaction					2752.7 (< 0.001)
No	31,929	72.5	12,110	27.5	
Yes	12,243	52.3	11,155	47.7	
Has an account in a bank/financial institution					2026.2 (< 0.001)
No	35,546	70.3	15,049	29.7	
Yes	8626	51.2	8215	48.8	
Use of Internet					398.2 (< 0.001)
Never	34,009	67.6	16,275	32.4	
Yes, last 12 months	9309	59.2	6419	40.8	
Yes, before last 12 months	854	59.9	571	40.1	
Respondent currently working					541.9 (< 0.001)
No	14,926	71.9	5836	28.1	
Yes	29,246	62.7	17,428	37.3	
Person who usually decides how to spend respondent’s earnings					2031 (< 0.001)
Respondent alone	14,375	66.7	7167	33.3	
Respondent & husband/partner	6726	47.7	7366	52.3	
Husband/partner alone	2882	82.3	618	17.7	
Someone else	46	88.5	6	11.5	
Owns land alone or jointly					246.6 (< 0.001)
Does not own	30,494	68.4	14,115	31.6	
Alone only	3449	63.2	2007	36.8	
Jointly only	7828	62.2	4767	37.8	
Both alone and jointly	1309	59.8	881	40.2	
Wealth index					1843.3 (< 0.001)
Poorest	4220	78.8	1132	21.2	
Poorer	6381	74.8	2147	25.2	
Middle	8602	71.4	3444	28.6	
Richer	11,328	65.5	5955	34.5	
Richest	13,641	56.3	10,587	43.7	
Residence					267.6 (< 0.001)
Urban	21,078	62.5	12,643	37.5	
Rural	23,094	68.5	10,622	31.5	
Age group					716.5 (< 0.001)
15–19	2355	82.0	518	18.0	
20–24	6928	70.9	2838	29.1	
25–29	10,087	67.6	4824	32.4	
30–34	8579	63.2	4991	36.8	
35–39	7300	61.1	4642	38.9	
40–44	5022	61.2	3184	38.8	
45–49	3902	63.2	2268	36.8	

### Multilevel logistic regression of factors associated with married women’s ability to make sexual and reproductive health decisions in sub-Saharan Africa

Table 4 portrays the outcomes of multilevel logistic regression, revealing the interconnected factors associated with the capacity of married women to engage in SRH decisions within SSA. When juxtaposed with married women in Benin (the referenced group), Madagascar (aOR = 4.95, 95% CI = 4.29–5.71), Uganda (aOR = 3.42, 95% CI = 2.99–3.90), Malawi (aOR = 2.84, 95% CI = 2.13–2.99), and Liberia (aOR = 2.53, 95% CI = 2.14–2.99) emerged as the top four SSA countries, showcasing proficiency in SRH decision-making. In contrast, Gambian, Guinean, and Malian married women, when compared to their Beninese counterparts, were less likely to partake in SRH decisions. Notably, South Africa ranked second in the hierarchy of countries where married women’s SRH decision-making was prominent, as revealed by Model 1; yet, it relinquished its position to sixth under Model V when education, digitalization, economic and demographic factors were introduced, while Madagascar consistently retained the top position.

**Table 4:** Multilevel logistic regression of factors associated with married women’s ability to make sexual and reproductive health decisions in sub-Saharan Africa

Variable	Model I	Model II	Model III	Model IV	Model V
	aOR (95% CI)	aOR (95% CI)	aOR (95% CI)	aOR (95% CI)	aOR (95% CI)
Country					
Benin, 2017	Ref				Ref
Burundi, 2016-17	1.92*** (1.68–2.19)				1.40*** (1.18–1.67)
Ethiopia, 2016	2.42*** (2.12–2.76)				2.04*** (1.69–2.45)
Gambia, 2019-20	0.72*** (0.64–0.81)				0.66*** (0.57–0.76)
Guinea, 2018	0.43*** (0.37–0.49)				0.51*** (0.43–0.61)
Liberia, 2019-20	3.78*** (3.30–4.32)				2.53*** (2.14–2.99)
Madagascar, 2021	8.57*** (7.62–9.65)				4.95*** (4.29–5.71)
Mali, 2018	0.15*** (0.12–0.18)				0.19*** (0.15–0.24)
Malawi, 2018	3.48*** (3.12–3.88)				2.84*** (2.13–2.99)
Nigeria, 2018	1.46*** (1.32–1.62)				1.06 (0.94–1.19)
Rwanda, 2019-20	4.25*** (3.78–4.77)				2.35*** (2.02–2.72)
Sierra Leone, 2019	1.26*** (1.11–1.41)				1.57*** (1.35–1.83)
Uganda, 2016	4.95*** (4.44–5.53)				3.42*** (2.99–3.90)
South Africa, 2016	5.17*** (4.58–5.84)				2.28*** (1.91–2.72)
Zambia, 2018	3.32*** (2.96–3.71)				1.46*** (1.26–1.68)
Zimbabwe, 2015	2.75*** (2.45–3.08)				1.27*** (1.13–1.42)
Interaction with education factors					
Education					
No education		Ref			Ref
Primary		3.55*** (3.36–3.74)			1.52*** (1.39–1.65)
Secondary		4.27*** (4.06–4.49)			1.97*** (1.81–2.13)
Higher		6.74*** (6.32–7.19)			2.37*** (2.13–2.64)
Knowledge of contraceptive method					
Knows no method		Ref			Ref
Knows only folkloric method		1.50 (0.34–6.69)			2.77 (0.55–3.88)
Knows only traditional method		3.45** (1.56–7.62)			2.10** (1.99–8.70)
Knows modern method		6.42*** (4.70–8.76)			3.19*** (1.99–5.13)
Interaction with digitalization factors					
Frequency of listening to radio					
Not at all			Ref		Ref
Less than once a week			1.18*** (1.13–1.24)		1.17*** (1.09–1.25)
At least once a week			1.33*** (1.28–1.39)		1.13*** (1.06–1.19)
Almost every day			1.43*** (1.22–1.66)		0.97 (0.79–1.20)
Frequency of watching television					
Not at all			Ref		Ref
Less than once a week			0.92*** (0.87–0.96)		1.14*** (1.06–1.22)
At least once a week			0.95** (0.91–0.99)		1.06 (0.99–1.14)
Almost every day			1.54*** (1.38–1.72)		1.056 (0.87–1.28)
Use mobile phone for financial transaction					
No			Ref		Ref
Yes			1.97*** (1.89–2.04)		1.21*** (1.14–1.28)
Has an account in a bank/financial institution					
No			Ref		Ref
Yes			1.77*** (1.70–1.85)		1.17*** (1.10–1.25)
Use of internet					
Never			Ref		Ref
Yes, last 12 months			0.95** (0.91–0.99)		1.16*** (1.08–1.24)
Yes, before last 12 months			1.06 (0.94–1.18)		0.97 (0.81–1.32)
Interaction with economic autonomy factors					
Respondent currently working					
No				Ref	Ref
Yes				1.23*** (1.12–1.35)	1.19*** (1.08–1.32)
Person who usually decides how to spend respondent’s earnings					
Respondent alone				Ref	Ref
Respondent & husband/partner				2.19*** (2.09–2.29)	1.29*** (1.22–1.36)
Husband/partner alone				0.43*** (0.39–0.47)	0.37*** (0.33–0.41)
Someone else				0.26* (0.11–0.61)	0.25** (0.09–0.63)
Owns land alone or jointly					
Does not own				Ref	Ref
Alone only				1.22*** (1.12–1.33)	1.08 (0.97–1.18)
Jointly only				1.15*** (1.09–1.22)	0.984 (0.92–1.05)
Both alone and jointly				1.23** (1.09–1.38)	0.97 (0.85–1.11)
Interaction with demographic factors					
Wealth index					
Poorest					Ref
Poorer					1.03 (0.90–1.17)
Middle					0.99 (0.87–1.12)
Richer					1.17** (1.04–1.33)
Richest					1.18** (1.04–1.35)
Residence					
Urban					Ref
Rural					0.95 (0.84–1.01)
Age group					
15–19					Ref
20–24					1.24** (1.02–1.50)
25–29					1.42*** (1.17–1.71)
30–34					1.65*** (1.37–1.99)
35–39					1.94*** (1.61–2.35)
40–44					1.90*** (1.57–2.31)
45–49					1.82*** (1.49–2.21)
Model X^2^	10354.171***	5626.901***	3853.449***	2099.301***	7886.305***
−2 Log Likelihood	76543.374	81270.644	83044.096	48323.335	42536.332
Nagelkerke R^2^	0.297	0.261	0.277	0.273	0.355
N	67,437	67,437	67,437	67,437	67,437

Significant at *p* <.05*,*p* <.01**,*p* <.001***

*Ref* Reference category, *CI* Confidence interval, *N* Weighted count, *%* Weighted percentage, *aOR* Adjusted Odds Ratio

Model I = Adjusted for country-level variable

Model II = Adjusted for women’s education variables

Model III = Adjusted for digitalization variables

Model IV = Adjusted for women’s economic autonomy variables

Model V = Adjusted for all individual and contextual variables, including age, wealth index, residence, and country

Education emerged as a significant influencer, amplifying married women’s SRH decision-making prowess in SSA. Women with higher education (aOR = 2.37, 95% CI = 2.13–2.64) displayed double the likelihood of making SRH decisions compared to those with no formal education. Likewise, a familiarity with modern contraceptive methods (aOR = 3.19, 95% CI = 1.99–5.13) tripled the probability of SRH decision-making among married women. The examination of digitalization’s impact disclosed that married women who listened to the radio at least once weekly (aOR = 1.13, 95% CI = 1.06–1.19) exhibited greater SRH decision-making involvement. Although initially significant, television’s influence waned in Model V. Those who watched television less than once per week (aOR = 1.14, 95% CI = 1.06–1.22) showed a heightened inclination towards SRH decision-making. Similarly, utilizing mobile phones for financial transactions (aOR = 1.21, 95% CI = 1.14–1.28), owning a bank/financial institution account (aOR = 1.17, 95% CI = 1.10–1.25), and using the internet within the past year (aOR = 1.16, 95% CI = 1.08–1.24) heightened the likelihood of engaging in SRH decisions.

Economic autonomy exhibited pronounced influence on married women’s SRH decisions. Those presently employed (aOR = 1.19, 95% CI = 1.08–1.32) were more likely to partake in SRH decisions, while women allowing their husbands to dictate expenditure (aOR = 0.37, 95% CI = 0.33–0.41) were 63% less likely to do so compared to self-decision-makers. Land ownership, when considered alone or jointly with husbands, initially amplified SRH decision-making (aOR = 1.22, 95% CI = 1.12–1.33; aOR = 1.15, 95% CI = 1.09–1.22), yet lost significance in Model V. Household wealth index exhibited a similar influence, favoring those from the wealthiest quintile (aOR = 1.18, 95% CI = 1.04–1.35) in contrast to the poorest households. Age emerged as a notable factor, peaking at 35–39 years (aOR = 1.94, 95% CI = 1.61–2.35) for SRH decision-making, gradually declining for women aged 40–44 (aOR = 1.90, 95% CI = 1.57–2.31) compared to those aged 15–19.

## Discussion

We investigated education, economic autonomy, and digitalization as factors intertwined with married women’s capacity to engage in SRH decisions within SSA. Our research spotlighted Madagascar, Uganda, Malawi, and Liberia as the top four nations in SSA where married women were more adept at making SRH decisions. Extensive studies underscored how governmental commitment and international interventions bolstered women’s SRH in these countries: Madagascar [37], Uganda [3], Malawi [6], and Liberia [4]. The sustained commitment of entities such as UNFPA [38], WHO [39], and USAID [40] to empowering women, granting them access to reproductive health commodities, supporting family planning services, and combating gender-based violence may have galvanized these advancements.

Comparatively, our findings revealed that married women from Gambia, Guinea, and Mali exhibited insubstantial SRH decision-making abilities when juxtaposed against their Beninese counterparts. Previous research echoed this by revealing lower contraceptive utilization among married women in these regions: Gambia [41], Guinea [42], and Mali [43]. While some countries excelled in SRH decisions among married women, the addition of various variables unveiled contrasting outcomes. For instance, South Africa emerged as the second-highest country for SRH decision-making ability, solely considering digital factors, yet fell to sixth position when education, economic considerations, and other factors were factored in. Research in South Africa identified issues such as inadequate contraceptive use, negative perceptions, and early sexual debut as influencing reproductive health decisions among its women [5, 44].

Analyzing the effect of women’s education on SRH decisions, our study showcased that education significantly bolsters married women’s SRH decision-making capacity within SSA. Specifically, women with higher education were twice as likely than those with no formal education to partake in SRH decisions. Corroborating studies in Nigeria [45], Ethiopia [46], and Rwanda [47] underscored the pivotal role of education in women’s SRH rights. Similarly, our findings illuminated that awareness of contraception amplifies married women’s SRH decision-making potential. Notably, those acquainted with modern contraceptive methods were thrice as likely as those who lacked awareness to engage in SRH decisions. This mirrored findings in Zambia [21] and Burundi [48], which linked knowledge of contraception to improved SRH rights, while highlighting the influence of socio-cultural norms on SRH decisions in Benin [49].

Digitalization’s sway on married women’s SRH decisions emerged through our study, revealing that women who listened to the radio were more prone to participating in SRH decisions. Although initially significant, television’s influence waned in Model V. This could be attributed to the inclusion of other variables, as studies pointed out that access to television might be hindered by financial constraints, particularly among rural women in SSA [50, 51]. Moreover, incorporating variables such as education and economic factors into the final model indicated that television’s effect on SRH decisions was less pronounced, highlighting the multifaceted nature of SRH decision-making among married women. Additionally, our study discovered that mobile phone use for financial transactions, bank account ownership, and internet utilization within the last year heightened married women’s likelihood of engaging in SRH decisions. Prior studies demonstrated the links between mobile phone access, internet use, and women’s reproductive health in various SSA countries [14, 52]. Furthermore, the interplay between women’s financial inclusion, bank account ownership, gender norm improvements, empowerment, and reproductive health was observed [53, 54].

Our results also spotlighted economic autonomy’s role in married women’s SRH decisions. Currently employed women were more inclined towards participating in SRH decisions compared to their non-working counterparts in Madagascar, Uganda, Malawi, Rwanda, and South Africa. This was consistent with previous findings that non-working women displayed lower likelihoods of engaging in reproductive health decisions within SSA [55]. Related research emphasized the interconnectedness of women’s employment, financial inclusion, and sexual autonomy [53, 54]. In alignment with our findings, women who permitted their husbands to dictate expenditure displayed diminished SRH decision-making capacities, underscoring the impact of patriarchal norms [30, 56]. While land ownership initially appeared to influence SRH decisions, its significance faded in Model V, highlighting the prominence of other factors. The study further revealed that the household wealth index significantly affected married women’s SRH decision-making prowess, favoring those from more affluent backgrounds. Additionally, age played a pivotal role, peaking in the 35–39 age group, after which it gradually declined while remaining higher than among women aged 34 or younger. Contrary to some studies [55], our analysis did not find a significant relationship between place of residence and reproductive health decision-making.

## Strengths and limitations

The strength of our study resides in its utilization of nationally representative samples from diverse SSA countries. Leveraging the most recent DHS data enabled an accurate depiction of the current landscape within the region. Employing trained field research assistants contributed to reducing potential biases and errors during data collection. The incorporation of a syntax command to weight the DHS dataset served to minimize under- and oversampling discrepancies, bolstering the study’s validity and enhancing its applicability across populations.

However, the study’s limitations must be acknowledged when interpreting its findings. Notably, the reliance on cross-sectional surveys conducted within a single timeframe curtailed the ability to track respondents’ evolving behaviors and reproductive health decisions over time, as would be feasible in longitudinal studies. The retrospective nature of some questions posed to married women could introduce biases stemming from forgetfulness. While the involvement of proficient field research assistants may have mitigated this concern, prudence should still be exercised in extrapolating the study’s outcomes. Another limitation of this study is the heterogeneity of the population analyzed, as married women within the reproductive age group across the 16 SSA countries are diverse in terms of socioeconomic status, cultural norms, and access to resources. However, this limitation was addressed by employing a multi-level analytical approach, which accounted for both individual- and country-level variations, ensuring that the findings reflect patterns and associations that are robust across different contexts rather than being driven by any single group. More so, due to the cross-sectional nature of DHS datasets, the study refrains from attributing causal relationships to the observed associations unveiled through our analysis. Consequently, the study remains rooted in correlation rather than causation, emphasizing the need for cautious interpretation.

## Conclusions

Our study concludes that married women in SSA countries such as Madagascar, Uganda, Malawi, Rwanda, South Africa, and Liberia exhibit greater decision-making autonomy compared to their counterparts in Gambia, Guinea, and Mali, where capacity for decision-making is lower. Countries like Ethiopia, Sierra Leone, Zambia, Zimbabwe, Burundi, Nigeria and Benin demonstrated moderate to low decision-making capacity. The empowerment of married women in these countries is significantly influenced by educational attainment and exposure to information about contraception. Access to mediums like radio and the internet, along with the utilization of digital tools such as mobile phones and bank accounts for financial transactions, collectively contributes to enhancing married women’s decision-making capabilities regarding their SRH. Variables related to economic autonomy, such as women’s participation in paid work and their autonomy in financial matters, whether independently or jointly with their husbands, also play a pivotal role in strengthening married women’s capacity to make informed decisions regarding their SRH.

It’s noteworthy that the Sustainable Development Goals (SDGs) agenda has set forth specific targets for both gender equality and SRH improvement by the year 2030 [8]. As governments, civil societies, and other relevant stakeholders earnestly work toward realizing these ambitious goals, it is imperative to direct focused attention toward augmenting married women’s access to education, digital technologies, and economic independence. These endeavors are not only aligned with global developmental objectives but also hold the potential to contribute significantly to advancing women’s rights and well-being across the SSA region.

## Data Availability

The datasets generated and/or analyzed during the current study are publicly available at the DHS Program repository: https://dhsprogram.com/data/available-datasets.cfm. However, due to an ongoing review of U.S. foreign assistance programs, the DHS Program is currently on pause, and data access for new users is temporarily restricted. The data supporting the findings of this study are available to existing DHS users and may also be obtained from the authors upon reasonable request and with permission from the DHS Program.

## Abbreviations

4IR: Fourth Industrial Revolution
aOR: adjusted Odds Ratio
CI: Confidence interval
DHS: Demographic and Health Survey
ICTs: Information and communication technologies
Ref: Reference category
SDGs: Sustainable Development Goals
SRH: Sexual and reproductive health
SSA: Sub-Saharan African
